# Late Presentation of a Perforated Marginal Ulcer Three Years After Mini Gastric Bypass: A Rare Entity

**DOI:** 10.7759/cureus.94541

**Published:** 2025-10-14

**Authors:** Zafar I Gondal, Yusuf Gunay, Aleena Chaudhary, Dima Al-Qaimari, Sanar Al-Qaimari, Rana T Hamada

**Affiliations:** 1 Department of General Surgery, Sheikh Khalifa Hospital, Fujairah, ARE; 2 College of Medicine, University of Sharjah, Sharjah, ARE

**Keywords:** abdominal pain, anastomotic leak, bariatric surgery complications, gastrojejunostomy, graham’s patch, helicobacter pylori, laparoscopic repair, mini gastric bypass, postoperative perforation, proton pump inhibitors

## Abstract

Marginal ulcers (MUs) are an uncommon complication following one-anastomosis gastric bypass (OAGB). They are often managed conservatively, but in some cases, they can progress to perforation. Such ulcer-related perforations are particularly rare and usually occur early in the postoperative period. We report the case of a middle-aged woman who presented with sudden, severe abdominal pain several years after undergoing OAGB. She had no identifiable active risk factors. Abdominal X-ray showed free air under the diaphragm, and computed tomography (CT) scan demonstrated extraluminal air and contrast near the gastrojejunostomy, findings interpreted as suspicious for perforation at the anastomotic site. Diagnostic laparoscopy confirmed a perforated marginal ulcer (PMU) at the anastomotic site, with the anastomosis itself remaining intact. The diagnosis was made intraoperatively based on visual inspection, revealing an ulcer at the anastomotic site with an apex measuring approximately 1.5 cm. No biopsy was obtained, as the ulcer margins appeared benign with no evidence of ischemia or malignancy. The defect was repaired with a laparoscopic Graham’s patch and omentopexy. The patient recovered well and returned to normal activity shortly after discharge. This case is notable for its delayed presentation, progression of a marginal ulcer to perforation at the gastrojejunal anastomosis, and the absence of additional modifiable risk factors apart from the inherent risk associated with the anastomosis itself. It underscores the importance of maintaining a high index of suspicion for late complications in post-bariatric patients and shows that timely minimally invasive surgery can achieve excellent outcomes.

## Introduction

Anastomotic leak is one of the most feared complications in gastrointestinal and bariatric surgery, carrying high morbidity and mortality due to risks of peritonitis, sepsis, and multi-organ failure. Although uncommon, with rates typically below 6%, its consequences are severe, making early recognition and prompt management essential [1,2]. In bariatric surgery, the incidence and underlying mechanisms of anastomotic leaks vary by procedure. Roux-en-Y gastric bypass (RYGB) is the most widely studied operation, with well-documented leak rates of 1.5%-6% [1,2]. In comparison, data on mini gastric bypass (MGB), also referred to as one-anastomosis gastric bypass (OAGB), are less extensive despite the procedure’s increasing global use.

Marginal ulcers (MUs), another recognized complication after OAGB, may present with similar clinical and radiologic features to an anastomotic leak but differ in pathophysiology. These ulcers arise at or near the gastrojejunostomy, often related to acid exposure or local ischemia, and may rarely progress to perforation, mimicking a leak on imaging despite preservation of anastomotic integrity. However, timing is key: anastomotic leaks typically occur early postoperatively, whereas perforated marginal ulcers (PMUs) can present months to years later, so late imaging findings often reflect ulcer perforation rather than true anastomotic disruption.

MGB/OAGB has gained traction over the past two decades as a technically simpler operation, associated with shorter operative times and comparable efficacy in terms of weight loss and metabolic outcomes when compared to RYGB [3]. However, like all bypass procedures, it is associated with unique complications, one of the most significant being marginal ulceration at the gastrojejunal anastomosis. The reported incidence of marginal ulcers following MGB/OAGB is approximately 2%-3% [3]. Although many of these ulcers present within the first year postoperatively, delayed presentations are also recognized and may occur years after the index operation.

A marginal ulcer is a mucosal defect occurring at or just distal to the gastrojejunal anastomosis, typically developing along the jejunal side where the mucosa is exposed to acidic gastric secretions and bile reflux. While most marginal ulcers are managed medically, in rare cases, they can progress to perforation. Ulcer perforation is a serious complication and is both life‑threatening and technically challenging to manage. A recent cohort study reported an incidence of marginal ulcer perforation of 0.98% following OAGB, with a median time to presentation of 19 months. The majority were treated with omental patch repair, although some patients required conversion to RYGB. Long-term follow-up demonstrated a risk of ulcer recurrence in a subset of patients [4]. Known risk factors for marginal ulceration include smoking, nonsteroidal anti-inflammatory drug (NSAID) use, *Helicobacter pylori* infection, and poor adherence to prophylactic proton pump inhibitors, but late complications can occur even in the absence of these factors [3,4]. This may be attributed to multifactorial mechanisms such as chronic mucosal ischemia at the anastomotic site, exposure to bile reflux or acidic gastric contents, mechanical tension or staple-line vulnerability, and nutritional deficiencies that impair mucosal healing. These factors can predispose to delayed ulceration and, in rare cases, perforation despite the absence of traditional risk factors.

With the expanding adoption of MGB/OAGB worldwide, it is important to recognize not only its benefits but also its potential long-term complications. Awareness of rare but serious sequelae such as delayed perforation secondary to marginal ulceration is critical for timely diagnosis and appropriate surgical intervention, ultimately improving patient safety and long-term outcomes. However, data on very late complications, such as perforated marginal ulcers occurring several years after OAGB, remain exceedingly limited in the literature, leaving an important gap in clinical understanding.

Here, we present a case that helps bridge this gap by documenting a rare delayed perforated marginal ulcer occurring 34 months after mini gastric bypass in a patient without known risk factors such as smoking, NSAID use, or *Helicobacter pylori* infection. The delayed presentation, subtle initial symptoms, and absence of these risk factors make this case both unusual and clinically important, underscoring the need for continued long-term vigilance and follow-up in post-bariatric patients.

## Case presentation

In late January 2025, a 58-year-old woman with no known comorbidities presented to the emergency department at Sheikh Khalifa Hospital, Fujairah, with sudden-onset severe generalized abdominal pain that began while she was on duty as a nurse manager at the same hospital. The patient described the pain as sharp and rated it as the most severe she had ever experienced. The pain persisted and progressively worsened over the following hours, prompting her presentation to the emergency department. It was neither radiating nor referred. She denied any associated symptoms such as fever, nausea, vomiting, hematemesis, melena, hematochezia, or any prior history of abdominal pain.

She had no history of gastrointestinal symptoms except occasional dyspepsia, for which she would take antacid syrups. She reported no history of significant medical disease and no regular medications. She underwent a mini gastric bypass in March 2022 for class II obesity (body mass index (BMI): 40 kg/m²) and had no immediate postoperative complications. The patient achieved her target weight loss within one year, with a current BMI of 23 kg/m². She had been taking regular multivitamin supplements and had remained smoke-free since March 2022.

On arrival, her vital signs were as follows: heart rate of 102 beats per minute, blood pressure of 124/56 mmHg, and oxygen saturation of 96% on room air. Laboratory investigations showed white blood cell count of 7.84 × 10⁹/L (normal), neutrophils of 86.2% (elevated), and C‑reactive protein of 163.85 mg/L (elevated), indicating an inflammatory process. *Helicobacter*​​​​​​ *pylori *testing that had been performed during routine follow-up after her bariatric surgery, over three years prior, was negative, and no repeat testing had been done since.

**Figure 1:**
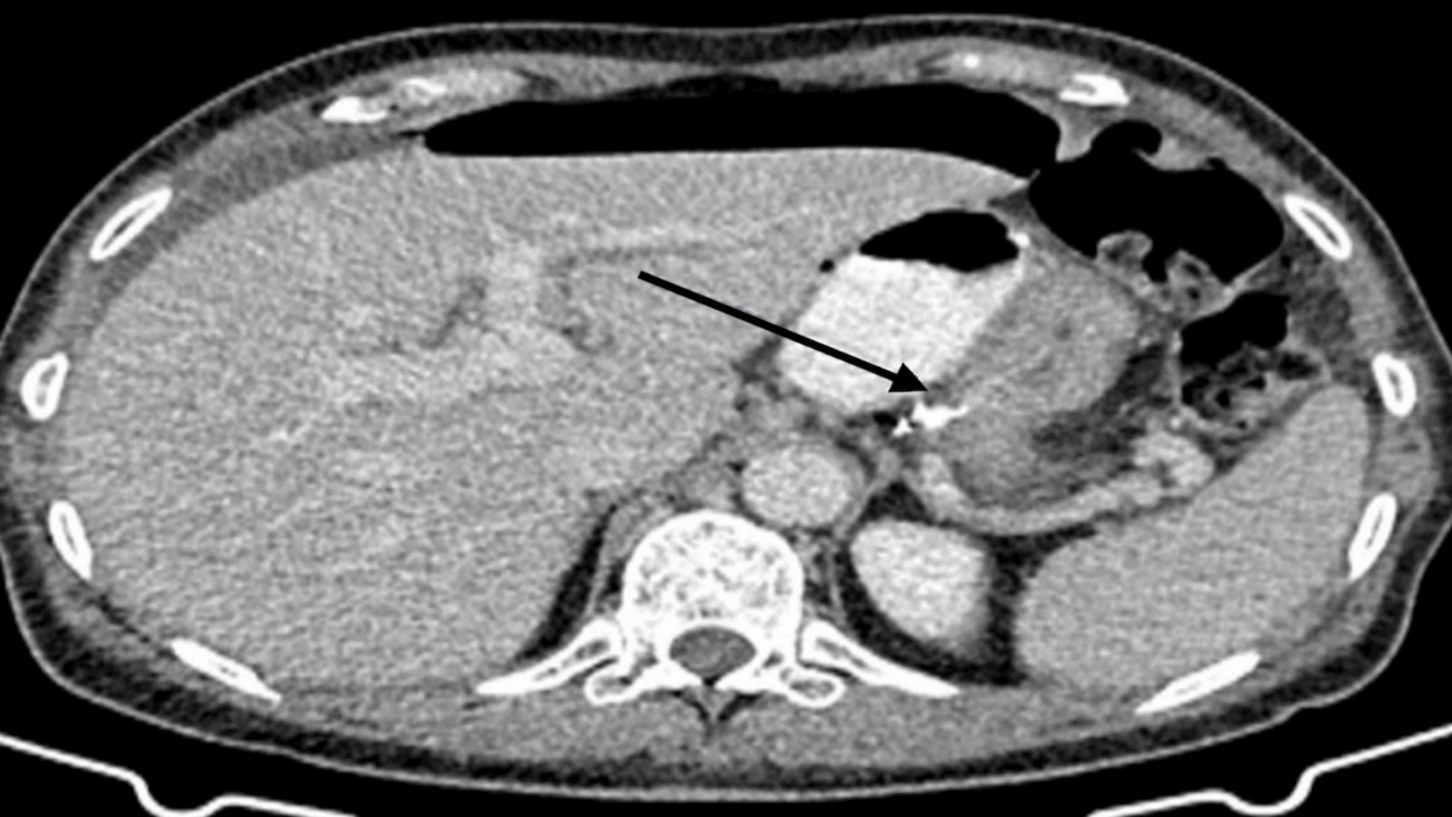
Oral contrast was visualized within the gastric and jejunal lumina, with extraluminal air and contrast at the gastrojejunostomy (arrow), findings consistent with perforation secondary to a marginal ulcer

The patient was promptly assessed in the emergency department. Examination revealed generalized abdominal tenderness with guarding. An initial abdominal X-ray demonstrated subdiaphragmatic lucency beneath the left hemidiaphragm, suggestive of pneumoperitoneum or interposed bowel. Subsequent computed tomography (CT) confirmed the presence of pneumoperitoneum, attributed to a suspected defect at the proximal gastrojejunal anastomosis, with multiple air locules noted in the surrounding area. Oral contrast was visualized within the gastric and jejunal lumina, with extraluminal air and contrast at the gastrojejunostomy, findings interpreted as suggestive of a perforation at the anastomotic site (Figure 1). An urgent surgical exploration was planned.

**Figure 2:**
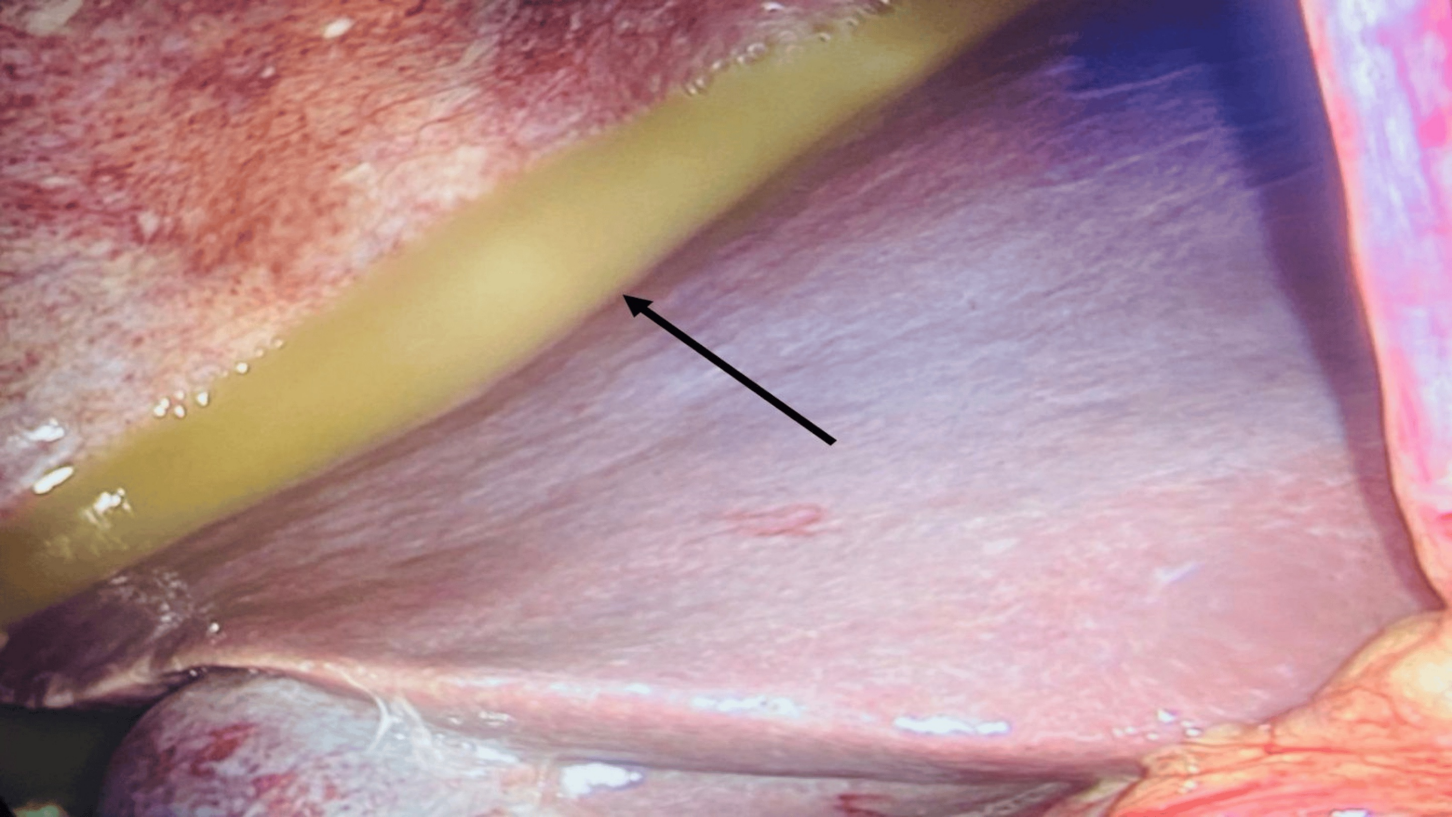
Diagnostic laparoscopy showing turbid, bile-stained fluid with loculations in the perihepatic space (arrow)

**Figure 3:**
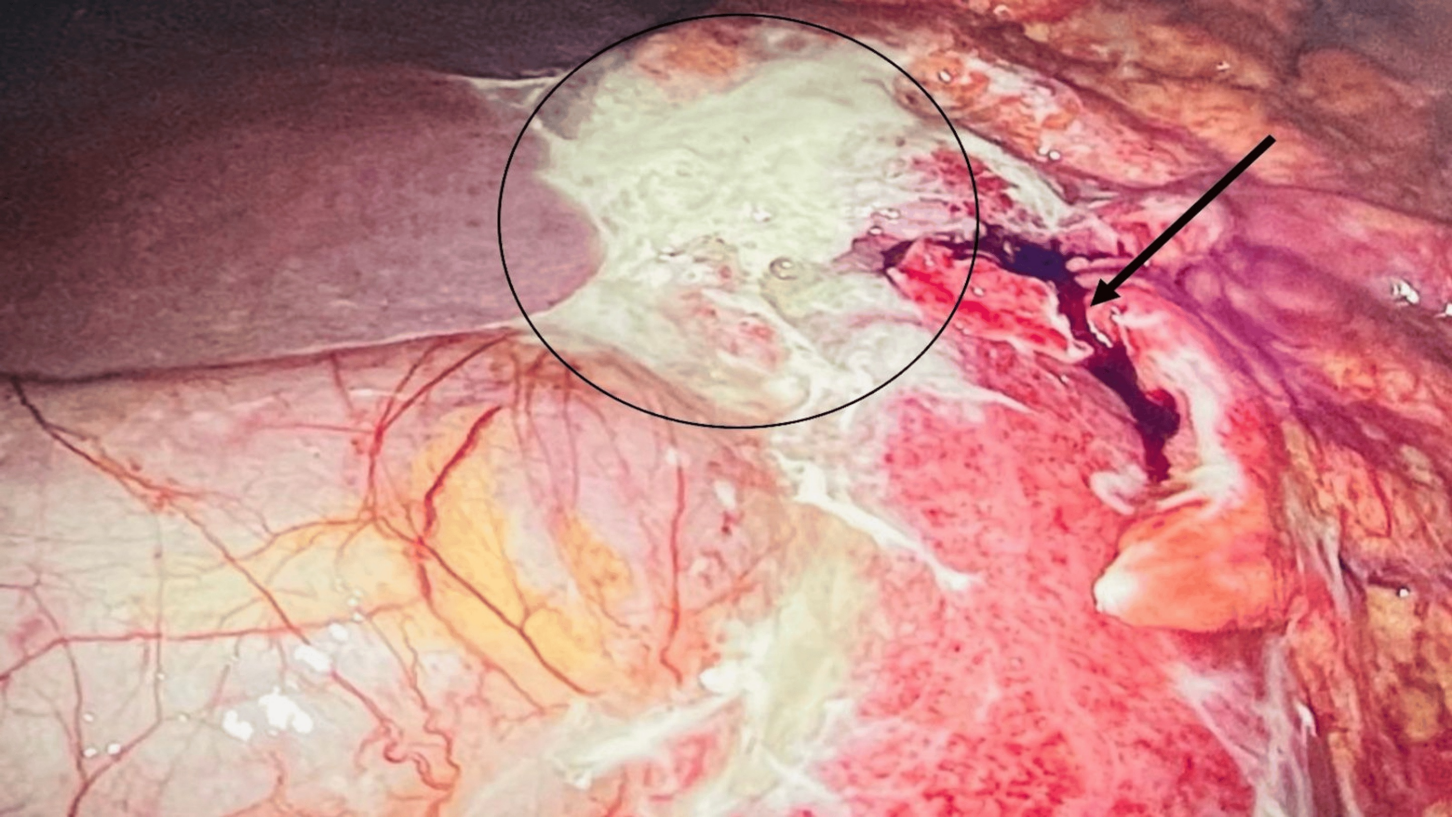
Diagnostic laparoscopy showing a perforated marginal ulcer at the anterior gastrojejunal anastomosis (arrow) and dense adhesions to the left lobe of the liver (circle)

During diagnostic laparoscopy, a perforation was identified at the gastrojejunal anastomosis, consistent with a perforated marginal ulcer causing localized tissue breakdown and partial dehiscence. Turbid, bile-stained fluid was present in the paracolic gutters, perihepatic and perisplenic areas, and pelvis, with noticeable loculations. Dense adhesions were also observed between the previous anastomosis and the left lobe of the liver. Closer inspection of the anastomotic site revealed ulceration at the gastrojejunal margin, suggesting a perforated marginal ulcer as the likely source, with an apex measuring approximately 1.5 cm. The assessment was made intraoperatively based on the gross appearance, and no tissue sample was taken since the margins appeared benign with no signs of ischemia or malignancy (Figure 2 and Figure 3).

Laparoscopic repair of the perforated marginal ulcer at the anastomotic site was performed using Graham’s patch with omentopexy, following thorough adhesiolysis and peritoneal washout. Two intra-abdominal drains were placed: one adjacent to the anastomotic site and another in the pelvic cavity to allow adequate drainage of peritoneal fluid and monitor for leakage. Drain output remained serosanguineous throughout the postoperative course. Although there were no signs of ongoing leakage or infection, the drains were kept in place until postoperative day (POD) 10 to ensure complete drainage and confirm anastomotic healing before removal.

Intraoperative endoscopy was performed to evaluate the stomach, gastrojejunostomy, and both proximal and distal limbs. Findings included a small gastric pouch and a gastrojejunostomy with a nasojejunal tube in situ. The endoscope was advanced smoothly across the anastomosis into the jejunal lumen without resistance, and no evidence of active leakage was observed.

The patient was transferred to the intensive care unit and was extubated uneventfully on POD 1. She was shifted to the surgical ward on POD 2. Due to the patient’s reluctance to take oral nutrition and to reduce postoperative nausea, total parenteral nutrition (TPN) was initiated on POD 4 via a peripherally inserted central catheter to ensure adequate nutritional support. Oral sips were introduced on POD 6 and gradually advanced to a full liquid/soft diet. Drain output remained serosanguineous throughout and was removed on POD 10.

The patient was discharged home in stable condition on POD 11. At follow-up, she demonstrated a smooth and uneventful recovery, tolerating a regular oral diet without dyspepsia or significant weight loss. She was able to resume work two weeks postoperatively. At outpatient follow-up, the patient continues under surveillance with plans for long-term endoscopic monitoring of the gastrojejunal anastomosis to detect recurrence. She was prescribed maintenance proton pump inhibitor therapy as ulcer prophylaxis and counseled on avoiding NSAIDs and smoking to reduce recurrence risk. No recurrence was noted at her most recent follow-up, but long-term monitoring remains ongoing.

## Discussion

Bariatric surgery remains a cornerstone intervention for the management of obesity and its related comorbidities, such as type 2 diabetes, obstructive sleep apnea, and hypercholesterolemia. These procedures primarily function by reducing gastric volume, altering gastrointestinal anatomy, or both; common examples include sleeve gastrectomy, biliopancreatic diversion, and gastric bypass techniques such as RYGB and MGB [5]. While effective, these surgeries are not without risk. Complications can arise in both early and late postoperative phases, often presenting with nonspecific symptoms such as abdominal pain. Although early complications are typically more acute and dramatic, late complications may be more insidious and require a high index of clinical suspicion [6].

Marginal ulcer (MU) formation is a well‑documented complication following gastric bypass procedures, particularly RYGB. However, its incidence following MGB/OAGB is significantly lower, at approximately 2%-3% [3]. In contrast, MU and its most severe manifestation (perforated marginal ulcer (PMU)) are far less common following MGB. A recent study reported a MU perforation incidence of 0.98% among 1,522 OAGB patients, with a median time to perforation of 19 months. The majority of these patients had one or more identifiable risk factors, such as smoking, NSAID or steroid use, or *Helicobacter pylori* infection. Most cases were successfully managed with laparoscopic omental patch repair, and only a single case of recurrence was documented during a median 28-month follow-up [4].

In the context of this case, our findings are both atypical and clinically significant. The patient presented with MU perforation 34 months after undergoing MGB, a timeframe notably longer than the median reported in existing literature. No routine postoperative endoscopy was performed, and therefore, we cannot definitively exclude the possibility that a gastric or anastomotic lesion was present and left untreated prior to this presentation. This distinguishes our case from others, where PMU typically occurs within two years of surgery. Although this patient lacked conventional risk factors such as smoking, NSAID use, or *H. pylori* infection, several mechanisms may have contributed to ulcer formation and eventual perforation. Dense adhesions observed during laparoscopy may have caused localized tension and mucosal ischemia at the anastomotic margin, predisposing to delayed ulceration. Chronic exposure of the jejunal mucosa to bile reflux from the long gastric pouch may also have contributed to mucosal injury. Additionally, subclinical micronutrient deficiencies and altered mucosal repair mechanisms after long-term weight loss could have impaired healing, rendering the anastomosis more vulnerable to breakdown over time.

Furthermore, her initial symptom of isolated abdominal pain without other systemic signs is consistent with the more subtle presentation patterns described in late-stage MU. Imaging confirmed the presence of pneumoperitoneum, and diagnostic laparoscopy revealed an anterior gastrojejunal ulcer perforation, consistent with the most common location noted in the literature [7]. The patient was treated successfully with laparoscopic Graham’s patch repair, the preferred intervention in approximately 59% of PMU cases, and had an uncomplicated postoperative course [8].

Similar cases of delayed perforation secondary to marginal ulceration after OAGB have been reported only sporadically in the literature. In most published series, perforation occurred within 12-24 months postoperatively and was associated with identifiable risk factors such as smoking, *H. pylori* infection, or poor adherence to proton pump inhibitors [4,7]. The interval of 34 months in our patient, in the absence of any known risk factors, therefore represents one of the latest reported presentations. Management strategies described in these reports, most commonly laparoscopic omental patch repair, align with our approach and demonstrate similarly favorable outcomes.

Despite surgical success, recurrence remains a concern in PMU management. Approximately 5% of patients may develop recurrent ulcers, underscoring the need for long-term surveillance and ongoing medical management. *Helicobacter pylori *infection remains an independent predictor of both initial ulcer formation and recurrence, further reinforcing the importance of preoperative screening and eradication. While proton pump inhibitors are widely accepted as first-line therapy for ulcer prevention and treatment, there is less agreement on whether refractory ulcers warrant surgical revision or extended conservative management [9-11].

Long-term management in such patients should include strict adherence to proton pump inhibitor therapy, avoidance of ulcerogenic medications, and regular surveillance endoscopy where feasible. Nutritional monitoring and patient education regarding early symptom recognition are also essential to prevent recurrence or delayed diagnosis of future ulceration.

We do not have definitive data on the exact onset of the ulcer or its duration before presentation. However, *H. pylori* testing had been performed during routine postoperative follow-up after her bariatric surgery and was negative. This finding reduces, but does not eliminate, the likelihood of *H. pylori*-related ulceration. Nevertheless, complications increase with untreated peptic ulcers, and the absence of early detection may have contributed to the severity of this presentation. Additionally, while the majority of the literature on marginal ulcers and perforation focuses on RYGB, cases of perforation caused by marginal ulceration following OAGB are particularly scarce, with only isolated reports and small series available [12,13]. Most OAGB-related marginal ulcers do not progress to perforation, and even fewer necessitate operative management, making the clinical course observed in our patient especially unusual.

## Conclusions

This case highlights that marginal ulcer perforation at the anastomotic site can occur several years after mini gastric bypass, even in the absence of traditional risk factors. Clinicians should maintain a high index of suspicion for late complications in post-bariatric patients presenting with acute abdominal pain, as early imaging, particularly computed tomography, remains the cornerstone of diagnosis. Prompt surgical intervention, ideally via minimally invasive techniques, can achieve favorable outcomes.

From a preventive perspective, long-term proton pump inhibitor therapy, strict avoidance of ulcerogenic medications, and routine *Helicobacter pylori* screening are key strategies to reduce marginal ulcer risk. Where feasible, periodic endoscopic surveillance and reinforcement of patient adherence to medical and nutritional follow-up are recommended to prevent recurrence. By emphasizing both diagnostic vigilance and preventive care, this case provides actionable lessons for the management of late post-OAGB complications.
